# Progress in Surgery

**Published:** 1896-04-11

**Authors:** 


### Progress in Surgery.

### SURGERY OF THE PANCREAS AND SPLEEN.

**Pancreatic Cyst.**—Richardson[1] publishes a case of a cyst as large as an adult head in a man twenty-six years of age, who had noticed a painless swelling in the upper part of the abdomen for six weeks. It was successfully treated by laparotomy, and free incision and drainage of the tumour after it had been stitched to the edges of the wound in the abdominal wall. The author, who found drainage effectual in two previous cases, states that many other observations confirm his opinion that complete healing takes place after this plan of treatment in most cases, and the danger is slight. This, therefore, should be the treatment in most cases. If the cyst can be easily separated from its attachments, and thoroughly extirpated without excessive hæmorrhage, it might be best to attempt such a procedure in view of the possible reaccumulation of fluid, with its remote dangers. The attempt at enucleation should be abandoned, however, as soon as it becomes evident that the cyst is so thoroughly incorporated with the surrounding pancreatic tissues, that it can be separated only by cutting.

**Splenopexis for Wandering Spleen.**—Knowing that the mortality of splenectomy for wandering spleen in forty-eight cases was 31 per cent., and moved by his objections to depriving the body of an important organ, Rydygier[2] attempted to obtain relief from the symptoms by securing the spleen in a stationary position. The patient, a woman, had symptoms of intestinal obstruction, and the spleen, enlarged and with a twisted pedicle, was found impacted in the pelvis. Rydygier cut a slit in the parietal peritoneum of the left hypochondrium opposite the ninth, tenth, and eleventh ribs, and excavated a pocket behind it, in which he placed the organ, the pocket being large enough to accommodate one half of its bulk. A flap of peritoneum was then cut out and turned over the still exposed portion of the spleen, allowing the pedicle to lie between the edge of the pocket and that of the flap. A few sutures were passed in order to make the result certain, but he feared to pass any deep sutures in the spleen. The patient made a good recovery. Plücker[3] also reports a case in a woman twenty-three years of age who had suffered for more than three years from pains in the stomach and over the sacrum caused by a firm, smooth tumour which occupied almost the whole of the smaller pelvis on the left side. After the abdomen had been opened in the median line, this tumour was found to be the spleen enlarged to about double its normal size. The wound in the anterior abdominal wall having been closed, the patient was turned over on her right side, and a second incision was made in the mid-axillary line from the costal arch to the crest of the ilium. The peritoneum having been exposed, an incision was made in this membrane, through which the movable spleen could be pressed through from the abdominal cavity. In this new and retro-peritoneal situation the displaced organ was fixed by sutures to the lumbar fascia, to the retro-peritoneal connective tissue and fat, and to the tenth rib. The edges of the peritoneal wound were very carefully stitched around the elongated pedicle of the spleen. The author prefers this method of dealing with a movable spleen to that of Rydygier described above, because he holds that: (1) It is less dangerous, and can be more readily performed. (2) There is no prolonged exposure of the peritoneal cavity, and the necessity of exposing and handling intestine is prevented. (3) The spleen is secured to unyielding and fixed structures, and finally there is much less risk of any subsequent tendency to hernia at the seat of operation.

**Splenectomy.**—Hahn[4] reports the case of a woman thirty-five years of age who had a fluctuating and movable tumour on the left side. On laparotomy being performed, the tumour was found to involve the spleen, which it was thought best to remove entirely. Examination of the tumour proved it to be a hydatid. Recovery was complete. The author refers to seven cases of this affection treated by splenectomy, of which five recovered. He warns against tapping, owing to the high mortality. If the removal of the tumour is prevented by firm adhesions, the wall may be stitched to the abdominal incision, and incised either immediately or at a second sitting. Sutton[5] also records three successful cases of splenectomy—one for enlarged spleen causing recurrent jaundice and vomiting by dragging on the stomach and kinking the duodenum and bile duct; one in a girl aged five years, for enlarged spleen with anæmia; and one for wandering spleen. They were removed by incision in the left linea semilunaris.

[1] Brit. Med. Journ., July 6th, 1895, and Internat. Med. Mag., July, 1895. [2] Amer. Med. and Surg. Bulletin, Sept. 15, 1895, and Wein. Klin. Woch., June 13th, 1895, No. 24, p. 431. [3] Brit. Med. Journ., Nov. 9, 1895, and Centralb. für Chir., No. 40, 1895. [4] Therapeut. Gazette, Sept. 16th, 1895, and Deut. Med. Woch., July 11th, 1895. [5] Lancet, Oct. 19th, 1895, p. 974.

